# Crystal structure of *N*-(*tert*-but­oxy­carbon­yl)glycyl-(*Z*)-β-bromo­dehydro­alanine methyl ester [Boc–Gly–(β-Br)^(*Z*)^ΔAla–OMe]

**DOI:** 10.1107/S1600536814025677

**Published:** 2014-11-29

**Authors:** Paweł Lenartowicz, Maciej Makowski, Bartosz Zarychta, Krzysztof Ejsmont

**Affiliations:** a Faculty of Chemistry, University of Opole, Oleska 48, 45-052 Opole, Poland

**Keywords:** crystal structure, β-bromo­dehydro­alanine, de­hydro­amino acid, non-helical conformation, hydrogen bonding

## Abstract

The title compound, C_11_H_17_BrN_2_O_5_, is a de­hydro­amino acid with a C=C bond between the α- and β-C atoms. The amino acid residues are linked *trans* to each other and there are no strong intra­molecular hydrogen bonds. The torsion angles indicate a non-helical conformation of the mol­ecule. The dipeptide folding is influenced by an inter­molecular N—H⋯O hydrogen bond and also minimizes steric repulsion. In the crystal, mol­ecules are linked by strong N—H⋯O hydrogen bonds, generating (001) sheets. The sheets are linked by weak C—H⋯O and C—H⋯Br bonds and short Br⋯Br [3.4149 (3) Å] inter­actions.

## Abstract 2

In a de­hydro­amino acid with a C=C bond between the α- and β-C atoms, the amino acid residues are linked *trans* to each other and there are no strong intra­molecular hydrogen bonds. The torsion angles indicate a non-helical conformation of the mol­ecule.

## Chemical context   

De­hydro­amino acids are analogues of amino acids characterized by the presence of an unsaturated doubled bound between the α- and β-carbon atoms in their structure. These compounds were found to be components of natural products (Bonauer *et al.*, 2006 ▶), with lanti­biotics being especially important since they are an important class of natural bacteriocins produced by Gram-positive bacteria (Willey & van der Donk, 2007 ▶). The development of synthetic methods for the preparation of de­hydro­peptides allows researchers to search for their practical applications and to use them as substrates for the production of peptidomimetics. One of the inter­esting classes of such mimetics are β-bromo-de­hydro­amino acids and their derivatives, which are usually obtained by radical halogenation of de­hydro­amino acids using *N*-bromo­succinimide (NBS). This reaction proceeds in two steps, namely by halogenation of de­hydro­amino acids, which gives α-bromo-imines, followed by tautomerization to the desired products upon treatment with an amine (Coleman & Carpenter, 1993 ▶; Zhang *et al.*, 2002 ▶). β-Bromo-de­hydro­amino acid derivatives are useful substrates in coupling reactions with alkynes (Singh *et al.*, 2003 ▶) or organoboranes (Collier *et al.* 2002 ▶; Zhang *et al.*, 2002 ▶). Further asymmetric hydrogenation of their double bound allows non-proteinogenic α-amino acids and their derivatives to be obtained. Another important reaction of β-bromo-α,β-de­hydro­amino acid derivatives in drug research is their coupling cyclization in which oxazole derivatives are produced (Liu *et al.*, 2014 ▶).
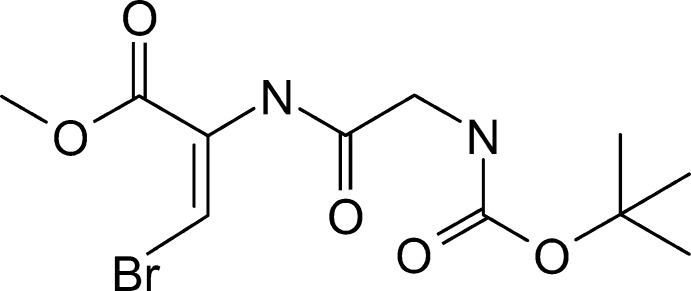


## Structural commentary   

**Figure 1:**
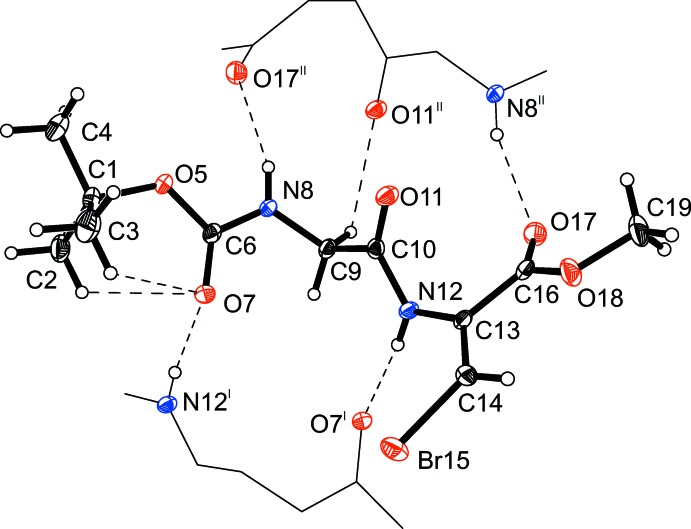
The mol­ecular structure of Boc–Gly–(β-Br)^(*Z*)^ΔAla–OMe along with selected intra­molecular hydrogen bonds (dashed lines), drawn with 50% displacement ellipsoids.

The mol­ecular structure of the title compound, (I), is shown in Fig. 1 ▶. The amino acids in the compound are linked *trans* to each other. The ω^2^ angle (C9—C10—N12—C13) is 175.79 (16)°, while ω^3^ (O5—C6—N8—C9) is 176.12 (15)°. There are no strong intra­molecular hydrogen bonds. The values of the ϕ^2,3^ and ψ^2,3^ angles corresponds to a non-helical conformation (Venkatachalam, 1968 ▶). The dipeptide folds accordingly to the inter­molecular N—H⋯O-type hydrogen bonds. The β-bromo-de­hydro­alanine moiety shows typical geometrical tendencies. The C10—N12 bond is longer [1.366 (2) Å] than a typical bond in alanine, while the N12—C13 bond is shorter [1.406 (2) Å]. This effect is common for other de­hydro-residues (Ajó *et al.*, 1979 ▶; Pieroni *et al.* 1975 ▶; Rzeszotarska *et al.*, 2002 ▶; Jain & Chauhan, 1996 ▶). This indicates conjugation between the side chain of de­hydro­alanine and the peptide bond. The torsion angles around the Br(H)C=C grouping are −0.9 (3) and −174.28 (13)° (N12—C13—C14—Br15 and C16—C13—C14—Br15, respectively), meaning the stereochemistry about the bond is especially planar. This is consistent with the nature of an *sp* ^2^-hybridized carbon on C13. The valance angles around the de­hydro­alanine group show some unusual values, especially N12—C13—C14 [124.27 (18)°], which may correspond to the presence of the bromine atom in the structure. The other angles are normal, as the backbone of the mol­ecule is folded to minimize steric repulsion. The Boc group features two short intra­molecular C—H⋯O contacts

## Supra­molecular features   

**Figure 2:**
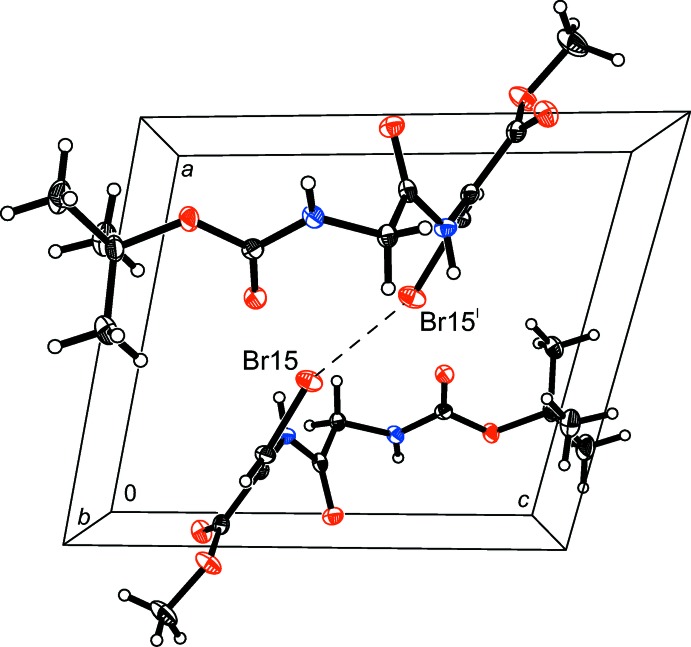
A packing diagram of (I), viewed along the *b* axis, showing the inter­molecular hydrogen-bonding scheme (dashed lines).

In the crystal, mol­ecules form two strong twin N—H⋯O (N8—H8*A*⋯O17^i^ and N12—H12*A*⋯O7^ii^) and one weak accompanying C9—H9*A*⋯O11^i^ hydrogen bonds (Fig. 1 ▶ and Table 1 ▶), forming infinite sheets in the (001) plane [symmetry codes: (i) −*x* + 2, −*y*, −*z* + 1 and (ii) −*x* + 3, −*y*, −*z* + 1]. The sheets are connected to each other by weak C14—H14*A*⋯O11^iii^ and C19—H19*B*⋯Br15^iii^ hydrogen bonds and one Br⋯Br^iv^ [3.4149 (3) Å] halogen bond (Fig. 2 ▶) of type I (Mukherjee & Desiraju, 2014 ▶) [symmetry codes: (iii) −*x* + 2, −*y* + 1, −*z* + 1; (iv) −*x* + 3, −*y* + 1, −*z* + 1].

## Synthesis and crystallization   

Boc–Gly–ΔAla and its methyl ester were prepared according to the methodology described by Makowski *et al.* (1985 ▶) and Cossec *et al.* (2008 ▶). The β-bromo-vinyl derivative was obtained based on a procedure described previously (Bull *et al.*, 2007 ▶). For this purpose 0.129 g (0.5 m*M*) of Boc–Gly–ΔAla–OMe was dissolved in 2.5 ml of di­chloro­methane and cooled to 193 K. Then, bromine 0.027 ml (0.5 m*M*) was added. The solution was stirred over 10 minutes followed by addition of tri­ethyl­amine 0.210 ml (1.5 m*M*). After 15 minutes, the mixture was quenched with 20 ml of saturated aqueous NaHCO_3_ and warmed to room temperature. The product was extracted by di­chloro­methane (3 × 15 ml). The organic layer was washed with brine (3 × 10 ml) and dried over anhydrous Na_2_SO_4_. Evaporation of the solvent at reduced pressure gave 0.119 g (0.35 m*M*) of crude product (70% yield). Recrystal­lization was performed from mixtures of diethyl ether/ethyl acetate­(2:1)/hexane solvents, yielding irregular colourless crystals. It is worth noting that in the case of our study, the formation of only the *Z* isomer was observed while in the preceding paper, the bromination of de­hydro­alanine-containing compound gave the *E* isomer. ^1^H NMR (400 MHz, DMSO) δ 1.38 (*s*, (*s*, 9H, C—H_3 *t*-Boc_), 3.67 (*s*, 3H, O—CH_3_), 3.69 (*d*, *J* = 6.2 Hz, 2H, C—H_2 Gly_), 7.05 (*t*, *J* = 6.2 Hz, 1H, N—H_Gly_), 7.30 (*s*, 1H, C=CHBr), 9.63 (s, 1H, N—H_β-Br–ΔAla_). ^13^C NMR (101 MHz, DMSO) δ 28.21, 42.79, 52.54, 78.12, 113.26, 132.88, 155.80, 162.63, 168.80. Melting point = 386–388 K.

## Refinement   

Crystal data, data collection and structure refinement details are summarized in Table 2 ▶. All H atoms were positioned geometrically and treated as riding on their parent C or N atoms: for methyl groups, C—H = 0.96 Å and *U*_iso_ (H) = 1.5*U*_eq_(C); for N atoms, N—H = 0.86 Å and *U*_iso_ (H) = 1.2*U*_eq_(C); for secondary C atoms, C—H = 0.97 Å and *U*_iso_ (H) = 1.2*U*_eq_(C), with no refinement of their parameters.

## Supplementary Material

Crystal structure: contains datablock(s) global, I. DOI: 10.1107/S1600536814025677/hb7312sup1.cif

Structure factors: contains datablock(s) I. DOI: 10.1107/S1600536814025677/hb7312Isup2.hkl

CCDC reference: 1035539

Additional supporting information:  crystallographic information; 3D view; checkCIF report

## Figures and Tables

**Table 1:** Hydrogen-bond geometry (, )

*D*H*A*	*D*H	H*A*	*D* *A*	*D*H*A*
C2H2*A*O7	0.96	2.51	3.058(2)	116
C3H3*A*O7	0.96	2.44	3.007(3)	117
N8H8*A*O17^i^	0.86	2.19	3.018(2)	162
C9H9*A*O11^i^	0.97	2.61	3.255(2)	124
N12H12*A*O7^ii^	0.86	2.04	2.901(2)	174
C14H14*A*O11^iii^	0.93	2.43	3.095(2)	129
C19H19*B*Br15^iii^	0.96	3.14	3.668(3)	117

Symmetry codes: (i) 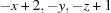; (ii) 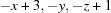; (iii) 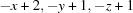.

**Table 2:** Experimental details

Crystal data	
Chemical formula	C_11_H_17_BrN_2_O_5_
*M*_r_	337.17
Crystal system, space group	Triclinic, *P* 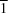
Temperature (K)	100
*a*, *b*, *c* ()	9.0431(4), 9.3160(4), 9.7540(4)
, , ()	83.381(3), 75.420(4), 64.863(4)
*V* (^3^)	719.92(6)
*Z*	2
Radiation type	Mo *K*
(mm^1^)	2.87
Crystal size (mm)	0.30 0.25 0.20

Data collection	
Diffractometer	Oxford Diffraction Xcalibur
Absorption correction	Multi-scan (*CrysAlis RED*; Oxford Diffraction, 2010 ▶)
*T*_min_, *T*_max_	0.655, 1.000
No. of measured, independent and observed [*I* > 2(*I*)] reflections	4860, 2780, 2490
*R*_int_	0.016
(sin /)_max_ (^1^)	0.617

Refinement	
*R*[*F* ^2^ > 2(*F* ^2^)], *wR*(*F* ^2^), *S*	0.024, 0.066, 1.06
No. of reflections	2780
No. of parameters	172
H-atom treatment	H-atom parameters constrained
_max_, _min_ (e ^3^)	0.53, 0.43

Computer programs: *CrysAlis CCD* and *CrysAlis RED* (Oxford Diffraction, 2010 ▶), *SHELXS2014* and *SHELXL2014* (Sheldrick, 2008 ▶), *SHELXTL* (Sheldrick, 2008 ▶).

## supplementary crystallographic information

### Crystal data

**Table:**

C_11_H_17_BrN_2_O_5_	*Z* = 2
*M**_r_* = 337.17	*F*(000) = 344
Triclinic, *P*1	*D*_x_ = 1.555 Mg m^−^^3^
*a* = 9.0431 (4) Å	Mo *K*α radiation, λ = 0.71073 Å
*b* = 9.3160 (4) Å	Cell parameters from 4860 reflections
*c* = 9.7540 (4) Å	θ = 3.2–26.0°
α = 83.381 (3)°	µ = 2.87 mm^−^^1^
β = 75.420 (4)°	*T* = 100 K
γ = 64.863 (4)°	Irregular, colourless
*V* = 719.92 (6) Å^3^	0.30 × 0.25 × 0.20 mm

### Data collection

**Table:**

Oxford Diffraction Xcalibur diffractometer	2780 independent reflections
Radiation source: fine-focus sealed tube	2490 reflections with *I* > 2σ(*I*)
Graphite monochromator	*R*_int_ = 0.016
Detector resolution: 1024 pixels mm^-1^	θ_max_ = 26.0°, θ_min_ = 3.2°
ω scan	*h* = −8→11
Absorption correction: multi-scan (*CrysAlis RED*; Oxford Diffraction, 2010)	*k* = −10→11
*T*_min_ = 0.655, *T*_max_ = 1.000	*l* = −12→12
4860 measured reflections	

### Refinement

**Table:**

Refinement on *F*^2^	0 restraints
Least-squares matrix: full	Hydrogen site location: inferred from neighbouring sites
*R*[*F*^2^ > 2σ(*F*^2^)] = 0.024	H-atom parameters constrained
*wR*(*F*^2^) = 0.066	*w* = 1/[σ^2^(*F*_o_^2^) + (0.0444*P*)^2^] where *P* = (*F*_o_^2^ + 2*F*_c_^2^)/3
*S* = 1.06	(Δ/σ)_max_ = 0.001
2780 reflections	Δρ_max_ = 0.53 e Å^−^^3^
172 parameters	Δρ_min_ = −0.43 e Å^−^^3^

### Special details

**Table:**

Experimental. CrysAlis RED, Oxford Diffraction Ltd., Version 1.171.33.57 (release 26-01-2010 CrysAlis171 .NET) (compiled Jan 26 2010,14:36:55) Empirical absorption correction using spherical harmonics, implemented in SCALE3 ABSPACK scaling algorithm.
Geometry. All e.s.d.'s (except the e.s.d. in the dihedral angle between two l.s. planes) are estimated using the full covariance matrix. The cell e.s.d.'s are taken into account individually in the estimation of e.s.d.'s in distances, angles and torsion angles; correlations between e.s.d.'s in cell parameters are only used when they are defined by crystal symmetry. An approximate (isotropic) treatment of cell e.s.d.'s is used for estimating e.s.d.'s involving l.s. planes.

### Fractional atomic coordinates and isotropic or equivalent isotropic displacement parameters (Å^2^)

**Table:**

	*x*	*y*	*z*	*U*_iso_*/*U*_eq_	
C1	1.2957 (3)	−0.1495 (2)	0.9892 (2)	0.0186 (4)	
C2	1.4783 (3)	−0.2662 (3)	0.9655 (2)	0.0248 (5)	
H2A	1.5455	−0.2243	0.8952	0.037*	
H2B	1.4904	−0.3651	0.9336	0.037*	
H2C	1.5146	−0.2830	1.0527	0.037*	
C3	1.2675 (3)	0.0136 (3)	1.0284 (2)	0.0291 (5)	
H3A	1.3374	0.0518	0.9572	0.044*	
H3B	1.2952	0.0083	1.1182	0.044*	
H3C	1.1520	0.0844	1.0348	0.044*	
C4	1.1870 (3)	−0.2140 (3)	1.1000 (2)	0.0313 (5)	
H4A	1.0720	−0.1387	1.1146	0.047*	
H4B	1.2228	−0.2316	1.1875	0.047*	
H4C	1.1975	−0.3122	1.0679	0.047*	
O5	1.23184 (17)	−0.14194 (16)	0.86239 (14)	0.0186 (3)	
C6	1.2959 (2)	−0.0915 (2)	0.7363 (2)	0.0144 (4)	
O7	1.40332 (16)	−0.03851 (15)	0.71486 (14)	0.0161 (3)	
N8	1.2267 (2)	−0.10813 (19)	0.63638 (17)	0.0155 (3)	
H8A	1.1573	−0.1522	0.6578	0.019*	
C9	1.2670 (2)	−0.0531 (2)	0.49289 (19)	0.0149 (4)	
H9A	1.2529	−0.1157	0.4283	0.018*	
H9B	1.3835	−0.0685	0.4688	0.018*	
C10	1.1567 (2)	0.1209 (2)	0.47430 (19)	0.0143 (4)	
O11	1.00831 (16)	0.18140 (15)	0.53245 (14)	0.0179 (3)	
N12	1.23628 (19)	0.20273 (18)	0.38410 (17)	0.0146 (3)	
H12A	1.3421	0.1556	0.3484	0.017*	
C13	1.1490 (2)	0.3623 (2)	0.34831 (19)	0.0136 (4)	
C14	1.1990 (2)	0.4770 (2)	0.3536 (2)	0.0164 (4)	
H14A	1.1341	0.5792	0.3266	0.020*	
Br15	1.39326 (2)	0.44202 (2)	0.41252 (2)	0.02292 (9)	
C16	1.0010 (2)	0.4006 (2)	0.2866 (2)	0.0163 (4)	
O17	0.97138 (18)	0.30286 (17)	0.24167 (15)	0.0210 (3)	
O18	0.90913 (18)	0.55685 (16)	0.28281 (16)	0.0239 (3)	
C19	0.7741 (3)	0.6065 (3)	0.2098 (3)	0.0324 (5)	
H19A	0.7155	0.7199	0.2129	0.049*	
H19B	0.6979	0.5594	0.2552	0.049*	
H19C	0.8193	0.5731	0.1130	0.049*	

### Atomic displacement parameters (Å^2^)

**Table:**

	*U*^11^	*U*^22^	*U*^33^	*U*^12^	*U*^13^	*U*^23^
C1	0.0226 (10)	0.0227 (10)	0.0121 (9)	−0.0104 (9)	−0.0065 (8)	0.0034 (8)
C2	0.0266 (11)	0.0256 (11)	0.0202 (11)	−0.0075 (9)	−0.0108 (9)	0.0066 (9)
C3	0.0397 (14)	0.0268 (12)	0.0204 (11)	−0.0129 (10)	−0.0070 (10)	−0.0014 (9)
C4	0.0369 (13)	0.0432 (14)	0.0172 (11)	−0.0222 (11)	−0.0058 (9)	0.0088 (10)
O5	0.0202 (7)	0.0248 (7)	0.0141 (7)	−0.0133 (6)	−0.0061 (6)	0.0077 (6)
C6	0.0131 (9)	0.0102 (9)	0.0158 (10)	−0.0021 (7)	−0.0029 (7)	0.0040 (7)
O7	0.0173 (7)	0.0166 (7)	0.0174 (7)	−0.0100 (6)	−0.0049 (5)	0.0028 (5)
N8	0.0169 (8)	0.0164 (8)	0.0167 (8)	−0.0105 (7)	−0.0063 (7)	0.0074 (7)
C9	0.0175 (9)	0.0136 (9)	0.0136 (9)	−0.0069 (8)	−0.0042 (7)	0.0038 (7)
C10	0.0187 (10)	0.0152 (9)	0.0126 (9)	−0.0091 (8)	−0.0065 (8)	0.0017 (7)
O11	0.0153 (7)	0.0149 (7)	0.0198 (7)	−0.0054 (6)	−0.0009 (6)	0.0035 (5)
N12	0.0120 (8)	0.0130 (8)	0.0169 (8)	−0.0050 (6)	−0.0019 (6)	0.0034 (6)
C13	0.0147 (9)	0.0143 (9)	0.0115 (9)	−0.0069 (8)	−0.0019 (7)	0.0031 (7)
C14	0.0137 (9)	0.0164 (9)	0.0191 (10)	−0.0061 (8)	−0.0049 (8)	0.0021 (8)
Br15	0.01942 (12)	0.02164 (12)	0.03230 (14)	−0.01022 (9)	−0.00996 (9)	−0.00138 (8)
C16	0.0181 (10)	0.0171 (10)	0.0130 (9)	−0.0083 (8)	−0.0032 (8)	0.0060 (8)
O17	0.0250 (8)	0.0209 (7)	0.0224 (8)	−0.0129 (6)	−0.0093 (6)	0.0028 (6)
O18	0.0222 (7)	0.0168 (7)	0.0346 (9)	−0.0061 (6)	−0.0164 (6)	0.0071 (6)
C19	0.0287 (12)	0.0290 (12)	0.0417 (14)	−0.0095 (10)	−0.0229 (11)	0.0157 (10)

### Geometric parameters (Å, º)

**Table:**

C1—O5	1.474 (2)	C9—C10	1.518 (2)
C1—C3	1.508 (3)	C9—H9A	0.9700
C1—C2	1.517 (3)	C9—H9B	0.9700
C1—C4	1.521 (3)	C10—O11	1.220 (2)
C2—H2A	0.9600	C10—N12	1.366 (2)
C2—H2B	0.9600	N12—C13	1.406 (2)
C2—H2C	0.9600	N12—H12A	0.8600
C3—H3A	0.9600	C13—C14	1.335 (3)
C3—H3B	0.9600	C13—C16	1.494 (3)
C3—H3C	0.9600	C14—Br15	1.8715 (19)
C4—H4A	0.9600	C14—H14A	0.9300
C4—H4B	0.9600	C16—O17	1.204 (2)
C4—H4C	0.9600	C16—O18	1.337 (2)
O5—C6	1.345 (2)	O18—C19	1.447 (2)
C6—O7	1.229 (2)	C19—H19A	0.9600
C6—N8	1.338 (2)	C19—H19B	0.9600
N8—C9	1.446 (2)	C19—H19C	0.9600
N8—H8A	0.8600		
			
O5—C1—C3	110.80 (16)	C9—N8—H8A	119.5
O5—C1—C2	109.75 (16)	N8—C9—C10	111.87 (15)
C3—C1—C2	112.96 (18)	N8—C9—H9A	109.2
O5—C1—C4	101.56 (15)	C10—C9—H9A	109.2
C3—C1—C4	110.90 (18)	N8—C9—H9B	109.2
C2—C1—C4	110.29 (18)	C10—C9—H9B	109.2
C1—C2—H2A	109.5	H9A—C9—H9B	107.9
C1—C2—H2B	109.5	O11—C10—N12	122.87 (17)
H2A—C2—H2B	109.5	O11—C10—C9	122.69 (16)
C1—C2—H2C	109.5	N12—C10—C9	114.41 (16)
H2A—C2—H2C	109.5	C10—N12—C13	121.43 (16)
H2B—C2—H2C	109.5	C10—N12—H12A	119.3
C1—C3—H3A	109.5	C13—N12—H12A	119.3
C1—C3—H3B	109.5	C14—C13—N12	124.27 (18)
H3A—C3—H3B	109.5	C14—C13—C16	118.50 (17)
C1—C3—H3C	109.5	N12—C13—C16	116.92 (16)
H3A—C3—H3C	109.5	C13—C14—Br15	123.17 (15)
H3B—C3—H3C	109.5	C13—C14—H14A	118.4
C1—C4—H4A	109.5	Br15—C14—H14A	118.4
C1—C4—H4B	109.5	O17—C16—O18	124.26 (18)
H4A—C4—H4B	109.5	O17—C16—C13	124.08 (18)
C1—C4—H4C	109.5	O18—C16—C13	111.61 (16)
H4A—C4—H4C	109.5	C16—O18—C19	115.57 (16)
H4B—C4—H4C	109.5	O18—C19—H19A	109.5
C6—O5—C1	121.71 (14)	O18—C19—H19B	109.5
O7—C6—N8	124.52 (17)	H19A—C19—H19B	109.5
O7—C6—O5	125.30 (17)	O18—C19—H19C	109.5
N8—C6—O5	110.18 (16)	H19A—C19—H19C	109.5
C6—N8—C9	120.96 (15)	H19B—C19—H19C	109.5
C6—N8—H8A	119.5		
			
C3—C1—O5—C6	62.8 (2)	C9—C10—N12—C13	175.79 (16)
C2—C1—O5—C6	−62.6 (2)	C10—N12—C13—C14	130.6 (2)
C4—C1—O5—C6	−179.35 (17)	C10—N12—C13—C16	−55.9 (2)
C1—O5—C6—O7	−4.7 (3)	N12—C13—C14—Br15	−0.9 (3)
C1—O5—C6—N8	174.87 (15)	C16—C13—C14—Br15	−174.28 (13)
O7—C6—N8—C9	−4.3 (3)	C14—C13—C16—O17	159.29 (19)
O5—C6—N8—C9	176.12 (15)	N12—C13—C16—O17	−14.6 (3)
C6—N8—C9—C10	−86.1 (2)	C14—C13—C16—O18	−18.4 (2)
N8—C9—C10—O11	−38.0 (3)	N12—C13—C16—O18	167.72 (16)
N8—C9—C10—N12	143.70 (16)	O17—C16—O18—C19	−4.6 (3)
O11—C10—N12—C13	−2.5 (3)	C13—C16—O18—C19	173.12 (17)

### Hydrogen-bond geometry (Å, º)

**Table:**

*D*—H···*A*	*D*—H	H···*A*	*D*···*A*	*D*—H···*A*
C2—H2*A*···O7	0.96	2.51	3.058 (2)	116
C3—H3*A*···O7	0.96	2.44	3.007 (3)	117
N8—H8*A*···O17^i^	0.86	2.19	3.018 (2)	162
C9—H9*A*···O11^i^	0.97	2.61	3.255 (2)	124
N12—H12*A*···O7^ii^	0.86	2.04	2.901 (2)	174
C14—H14*A*···O11^iii^	0.93	2.43	3.095 (2)	129
C19—H19*B*···Br15^iii^	0.96	3.14	3.668 (3)	117

Symmetry codes: (i) −*x*+2, −*y*, −*z*+1; (ii) −*x*+3, −*y*, −*z*+1; (iii) −*x*+2, −*y*+1, −*z*+1.
